# Wrinkle Structured Network of Silver-Coated Carbon Nanotubes for Wearable Sensors

**DOI:** 10.1186/s11671-019-3186-5

**Published:** 2019-11-29

**Authors:** Zhongyun Yuan, Zhen Pei, Muhammad Shahbaz, Qiang Zhang, Kai Zhuo, Chun Zhao, Wendong Zhang, Xingyi Ma, Shengbo Sang

**Affiliations:** 1 MicroNano System Research Center, Key Laboratory of Advanced Transducers and Intelligent Control System of Ministry of Education and Shanxi Province, College of Information & Computer Engineering, Taiyuan University of Technology, Taiyuan, 030024 China; 2 College of Information and Communication, Sungkyunkwan University, Chunchun-Dong, Changan-Ku, Suwon, 440-746 Korea; 3 Institute of Convergence Chemical Engineering Systems, Korea University, Seoul, 136713 Korea

**Keywords:** Soft-strain sensor, PDMS, Hydroxyl-functionalized CNT, Ag@CNT, Breath detection, Movement recognition

## Abstract

Soft-strain-based sensors are being increasingly used across various fields, including wearable sensing, behavior monitoring, and electrophysiological diagnostics. However, throughout all applications, the function of these sensors is limited because of high sensitivity, high-dynamic range, and low-power consumption. In this paper, we focus on improving the sensitivity and strain range of the soft-strain-based sensor through structure, surface, and sensitive unit treatment. Nanosilver (Ag)-coated hydroxyl-functionalized multi-walled carbon nanotubes (OH-f MWCNTs) were explored for highly acute sensing. With stretching and depositing methods, Ag@OH-f MWCNTs and polydimethylsiloxane (PDMS) are fabricated into a wrinkled and sandwich structure for a soft-strain-based sensor. The electronic properties were characterized in that the gauge factor (GF) = ΔR/R0 was 412.32, and the strain range was 42.2%. Moreover, our soft-strain-based sensor exhibits features including flexibility, ultra-lightweight and a highly comfortable experience in terms of wearability. Finally, some physiological and behavioral features can be sampled by testing the exceptional resistance change, including the detection of breath, as well as facial and hand movement recognition. The experiment exhibits its superiority in terms of being highly sensitive and having an extensive range of sensing.

## Introduction

Sensors play a vital role in medical diagnostics, particularly flexible sensors, which provide feasibility and stretchability for long-term health monitoring applications [1–8]. In recent years, soft strain sensors have broadened the range of applications for flexible sensors, such as cochlear implants [9], cerebral cortex control prostheses [10], electronic skin touch [11], as well as a variety of other applications [12–16]. Therefore, it is critical to improve the performance in terms of selectivity, sensitivity, and response to meet the requirements of advanced healthcare applications.

The continuous development of wearable flexible strain sensors has recently become more popular [17–23]. Silver (Ag) nanomaterials and carbon nanomaterials have attracted the interest of researchers owing to their excellent electrical and mechanical properties, low cost, and high stability [16, 24–26]. For example, strain sensors having a sandwich structure coupled with being laminated by an Ag nanowire network and a polydimethylsiloxane (PDMS) elastomer exhibit conscious characteristics of electrical conductivity and sensitivity [16, 24–26]. Strain sensors based on carbon nanomaterials have unique transparency and stability [20, 27–29]. These sensors can also be optimized by integrating the advantages of Ag and carbon nanomaterials. A sandwich structure strain sensor with graphene/Ag nanoparticle (AgNP) synergistic conductive network was prepared, where the AgNP was formed in situ, and graphene nanosheets were used as conductive bridges between them to ensure excellent initial conductivity and high wearability of the wearable sensor stretchability [30]. Shuqi Liu et al. prepared a flexible strain sensor with a conductive composite layer by pouring liquid PDMS onto polymer microspheres, a mixture of graphene oxide, and Ag nanowires [31]. However, the combination of Ag and carbon nanomaterials reduces the stretchability of the final nanocomposite, limiting its practical application in soft strain sensors. According to our previous studies using carbon nanotubes (CNTs) and AgNPs [32], the decrease in stretchability is related to the low binding energy between Ag and carbon nanomaterials.

In this work, we used OH-f MWCNTs to increase the binding energy between CNTs and Ag [33], and built a soft strain sensor based on the newly developed Ag@OH-f MWCNT nanocomposite using a brand new wrinkled structure design. The combination of binding energy and structure makes the soft change sensitive and less resistant. Ag@OH-f MWCNT nanocomposites were prepared by directly reducing nanosilver particles on the surface of CNTs. The components were confirmed and their morphology was characterized by scanning electron microscopy (SEM) and transmission electron microscopy (TEM). Soft strain sensors with wrinkled and sandwich structures were prepared by pre-stretching, surface treatment, and deposition methods using nanocomposites and PDMS. The electronic characteristics and piezoresistive effects of the sensor were analyzed. Finally, the sensor was applied to a portable respiratory detector and tested in facial expression recognition.

## Methods Section

### Synthesis and Characterization of Ag@OH-f MWCNTs

The Ag@OH-f MWCNT composite was obtained via simple reduction method. First, 0.5 mg OH-f MWCNTs (purchased from Chengdu Organic Chemicals Co. Ltd.) was dispersed in 300 mL silver nitrate aqueous solution (AR, 3 × 10^-2^ M). The mixture was then heated at 120 °C with magnetic stirring in oil bath. Next, 10 mL sodium citrate aqueous solution (AR, 1 wt%) was added into the mixture. Finally, the mixture was heated with further stirring for 1 h.

Characterization of Ag@OH-f MWCNTs was carried via scanning electron microscopy (SEM) and transmission electron microscopy (TEM). SEM images of the product were taken with a scanning electron microscope (SEM, JEOL S4700, Japan). TEM observations were performed on a JEOL JEM-1200EX (Japan) electron microscope.

### Preparation of Sensors

**Fig. 1:**
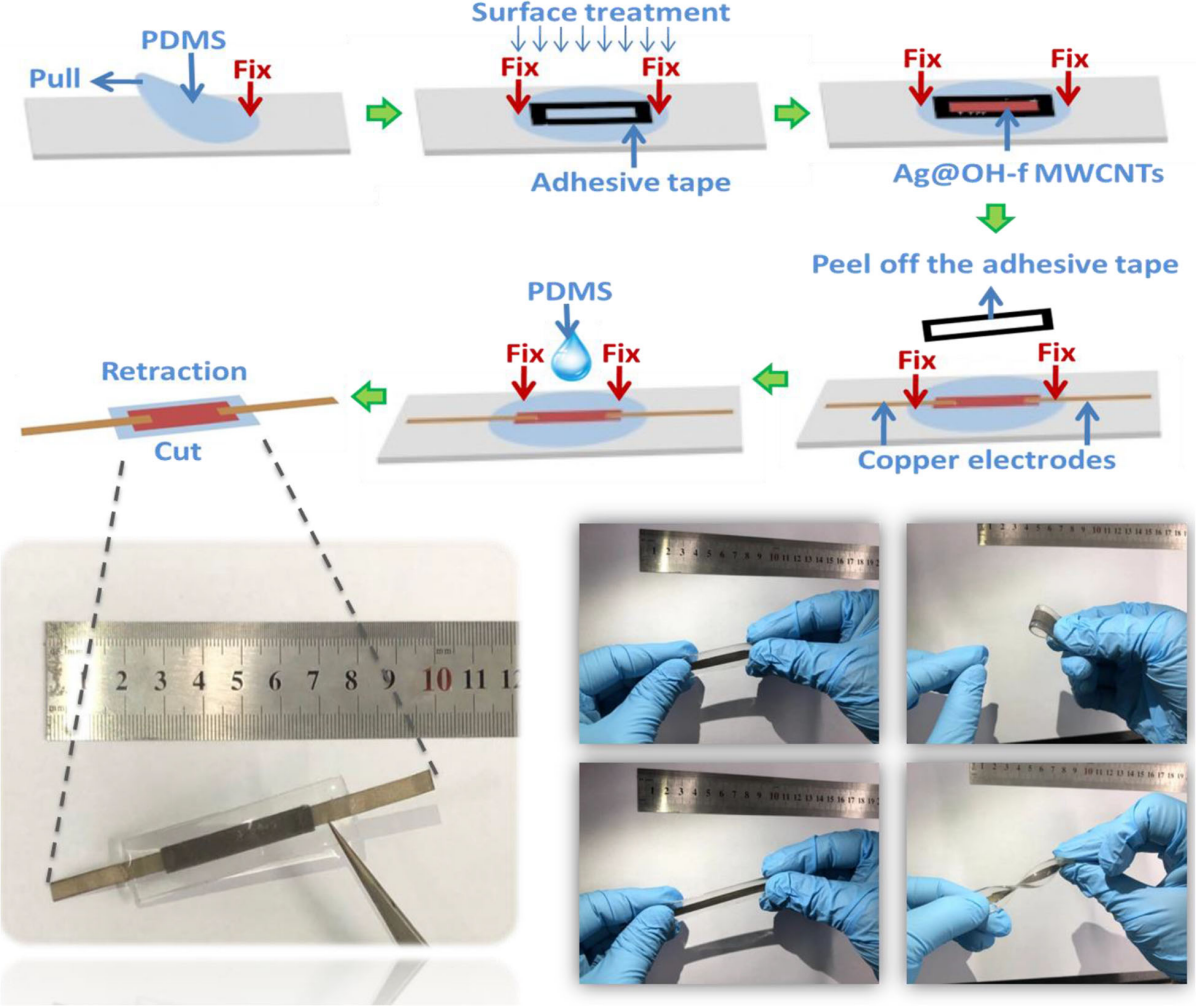
Fabrication process flowchart of the sensor

The fabrication flowchart of the sensor is shown in Fig. 1. PDMS film was obtained via degassing and heating (75 °C for 1 h) the mixture of PDMS elastomer and cross-linker. The PDMS film was peeled off and stretched to 110%, which was fixed by an adhesive tape with a rectangular hole. After the surface was treated with Schwarze P3C for 300 s, the Ag@OH-f MWCNT solation was dropped into the rectangular hole of the stretched PDMS film. The adhesive tape was then removed, and two copper electrodes were pasted on top of the nanocomposites. The PDMS solution was dropped on the top and heated to 75 °C for 1 h to strengthen the connection between the nanocomposites and electrodes. The Ag@OH-f MWCNT-based soft strain sensor with wrinkled structure was obtained after the pre-stress was released. The sensor obtained without surface treatment was prepared for comparison.

### Sensing Measurement

To study the current-voltage characteristics, I-V curves of the sensor were measured via digital oscilloscope (keithley2400) at room temperature. Two ends of the sensor were attached to the motorized moving stages (Zolix TSM25-1A and Zolix TSMV60-1s), and the resistance of the sensor was measured. Strain-sensing characteristics were tested by controlling the motorized stages moving.

### Application Measurement

The portable breathing detector was set up to verify the Ag@OH-f MWCNT-based soft strain sensor, which can be obtained by connecting and packaging the circuit. The detector was then tested with the sensor contacting the belly of the volunteer. The facial expression recognition of this sensor was measured via contacting the sensor on different parts of volunteer’s face.

## Results and Discussion

**Fig. 2:**
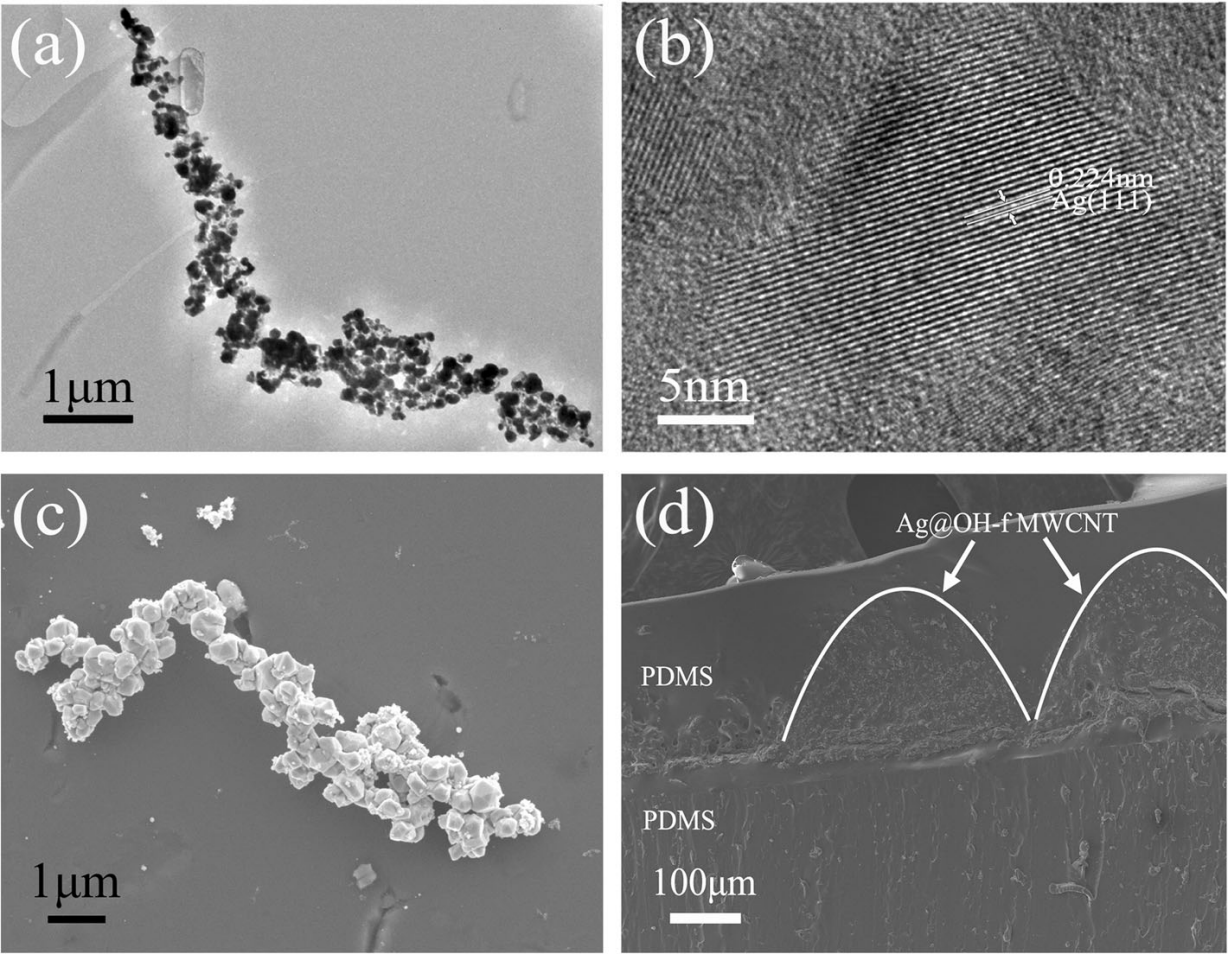
**a** TEM image of Ag@OH-f MWCNTs. **b** HRTEM image of Ag@OH-f MWCNTs. **c** SEM image of Ag@OH-f MWCNTs. **d** Cross-section SEM image of Ag@OH-f MWCNT-based soft strain sensor with wrinkled structure

The morphology of the Ag@OH-f MWCNT nanocomposite and the cross section of the sensor were characterized by SEM and TEM. The length and diameter of the CNTs are 1.25 ± 0.75 μm and 40 ± 10 nm, respectively. Ag was coated on the CNTs after synthesis, as shown in the TEM image (Fig. 2a). A high-resolution transmission electron microscope (HRTEM) image was taken and the crystallization lattice was clearly observed (Fig. 2b). The lattice space of 0.224 nm indicated the lowest surface energy during Ag crystallization in the direction of (111). The morphology of the nanocomposite is shown in the SEM image (Fig. 2c). In the synthetic process, the silver ions of AgNO_3_ were electrostatically concentrated by the hydroxyl groups of OH-f MWCNTs and followed by reduction into Ag atoms. The atoms crystallized along the CNTs and finally formed bulging necklace-like nanocomposites with the diameter of 200 ± 100 nm.

**Fig. 3:**
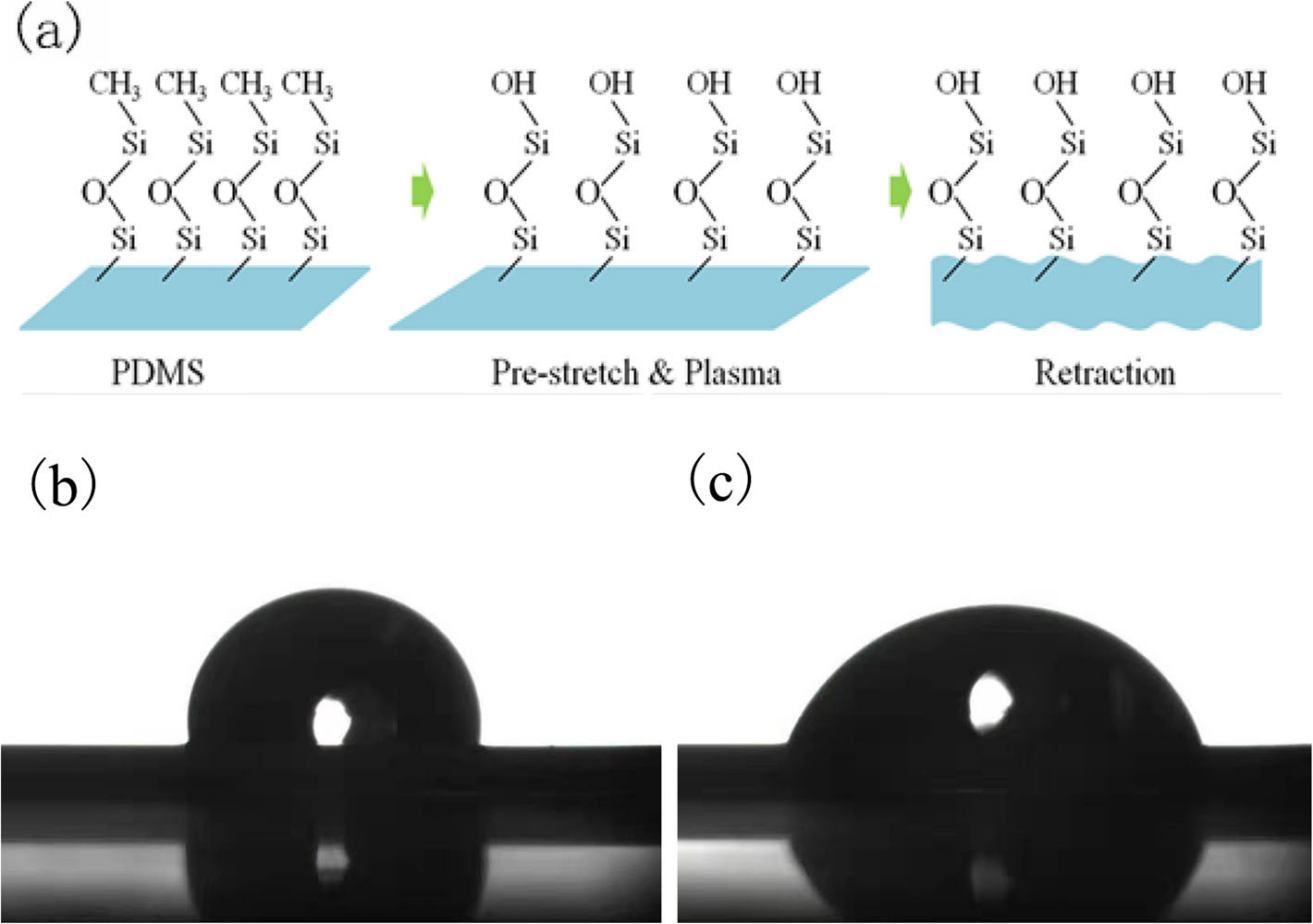
Water contact angle of PDMS (**a**) before and (**b**) after oxygen plasma surface treatment. **c** Schematic model of PDMS pre-stretching and oxygen plasma surface treatment

The PDMS was pre-stretched before surface treatment, and the wrinkled structure was obtained after the PDMS was loosened, as shown in Fig. 3a. The surface treatment of the PDMS was performed by high-energy oxygen plasma. The end of the molecular chain was changed from –Si–CH_3_ into –Si–OH, and thus the PDMS surface was converted from hydrophobic into hydrophilic [34]. Figure 3b and c demonstrates that the water contact angles of PDMS before and after oxygen plasma surface treatment were 91.6 ° and 47.9 °, respectively. The improvement of hydrophilicity increased the binding affinity between the PDMS and the nanocomposite.

After the combination of the PDMS and the nanocomposite, another PDMS layer was added to cover the top side, preventing the nanocomposite from denaturing or detaching. The Ag@OH-f MWCNT nanocomposite with wrinkled structure in the interlayer was demonstrated by SEM, as shown in Fig. 2d. The formation of the wrinkle layer transforms the necklace-like nanocomposite layer from a plane to a three-dimensional structure. When the sensor is deformed by external stress, the wrinkles will stretch back and the nanomaterial layer will continue to be stretched, thus expanding the stretch range and achieving stable sensing in this work.

**Fig. 4:**
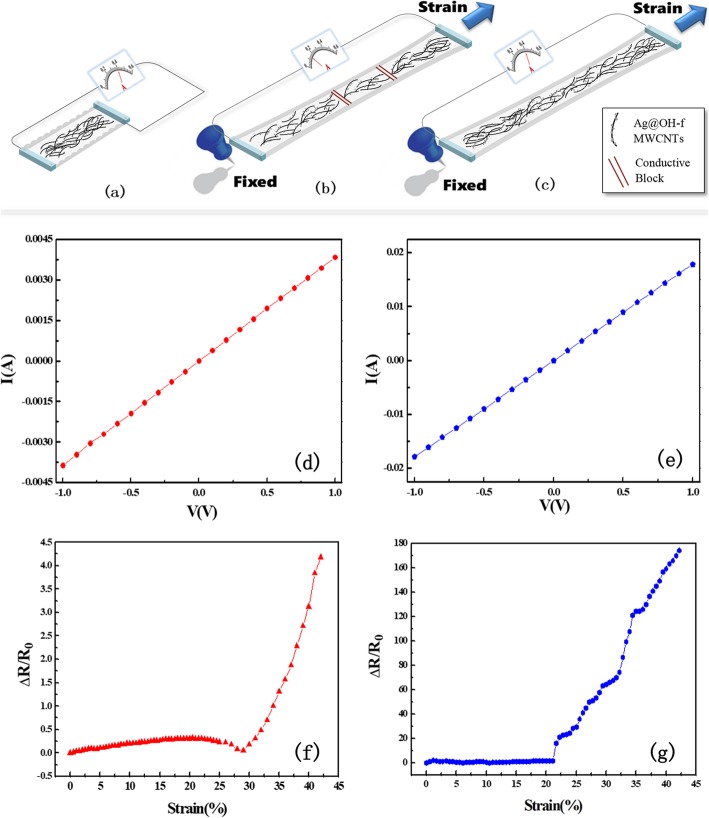
Stretch test of the soft strain sensor. **a**, (**b**), and (**c**) Schematic sensing model; I-V curves of (**d**) Ag@OH-f MWCNT-based soft strain sensor without wrinkled structure and (**e**) Ag@OH-f MWCNT-based soft strain sensor with wrinkled structure. The relative resistance changes of the sensors with (**f**) Ag@OH-f MWCNT-based soft strain sensor without wrinkled structure and (**g**) Ag@OH-f MWCNT-based soft strain sensor with wrinkled structure

Interestingly, the conductivity of the wrinkled structure was significantly enhanced in comparison with a flat structure, as characterized by current-voltage measurements under room temperature (Fig. 4d and e). Both of the sensors exhibited ohmic behavior, and the resistances of the sensors with the flat structure and the wrinkled structure were calculated as 256.41 Ω and 53.13 Ω, respectively. We suggest that the amount of Ag@OH-f MWCNTs, which is the key factor of sensor conductivity, was 4.8 times higher in the wrinkled structure than in the flat one.

Relative resistance change is one of the key parameters used to evaluate the performance of a soft strain sensor. As such, the relative resistance changes of the Ag@OH-f MWCNT-based strain sensor were then investigated, as shown in Fig. 4f and g, where ΔR and R0 represent the relative resistance change under deformation and the initial resistance of the sensor, respectively. The relative resistance change for the flat sensor is 4.18 at the maximum strain of 42% to the sensor (Fig. 4f), while it is 174 for the wrinkled sensor (Fig. 4g). Moreover, for the flat sensor, the resistance changed in the strain over 30%, whereas for the wrinkled one it changed in the strain over 20%. The resistance changes occurred when the configuration of the Ag@OH-f MWCNT networks in the PDMS began to change under the stretching strain. A stronger strain separated the networks with a larger spacing of the nanocomposite, decreasing the tunneling channels and the number of conductive paths. Further, we defined L0 as the initial length and ΔL as the relative elongation under axial strain to the sensor. Therefore, the gauge factor (GF) of the sensor could be calculated by the equation: $\mathrm{GF}=\frac{\Delta \mathrm{R}/{\mathrm{R}}_0}{\Delta \mathrm{L}/{\mathrm{L}}_0}$. The GF of the strain sensors with the flat and wrinkled structures was 9.95 and 412.32, respectively. GF is an indicator of sensitivity for soft strain sensors. Compared with the flat structure, the over 40-fold GF achieved by the wrinkled sensor manifested the design of our nanocomposite and was effective for further sensing applications.

We then proposed a model to understand the resistance variations of the strain sensors with wrinkled structure in the stretching process, as shown in Fig. 4. Figure 4a represents the Ag@OH-f MWCNT-based conductive networks inside the soft strain sensor with the free-state wrinkled structure. The surface treatment of PDMS to improve the binding affinity between the nanocomposite and PDMS was essential for the configuration and, consequently, for the performance of the sensor. Without the treatment, the binding of the nanocomposite to the hydrophobic PDMS was poor, the networks were easily disrupted, and the conductive paths were cut off by stretching (Fig. 4b). Therefore, the resistance of the sensor suddenly increased, which was caused by the tunneling channels and conductive paths number sharply decreasing, finally resulting in a small analytical range of sensing and low sensitivity. On the contrary, after oxygen plasma surface treatment, the hydrophilic PDMS showed high affinity to the nanocomposite (Fig. 4c). As demonstrated in Fig. 4d, the tunneling channels and conductive path number reduced gradually as the Ag@OH-f MWCNT networks were continuously separated by stretching. The related resistance change of the sensor with the surface treatment of PDMS was 41.63 times larger than without the surface treatment, suggesting that the surface treatment plays an important role in improving the sensitivity and strain range of the sensor based on the new Ag@OH-f MWCNT composite.

**Fig. 5:**
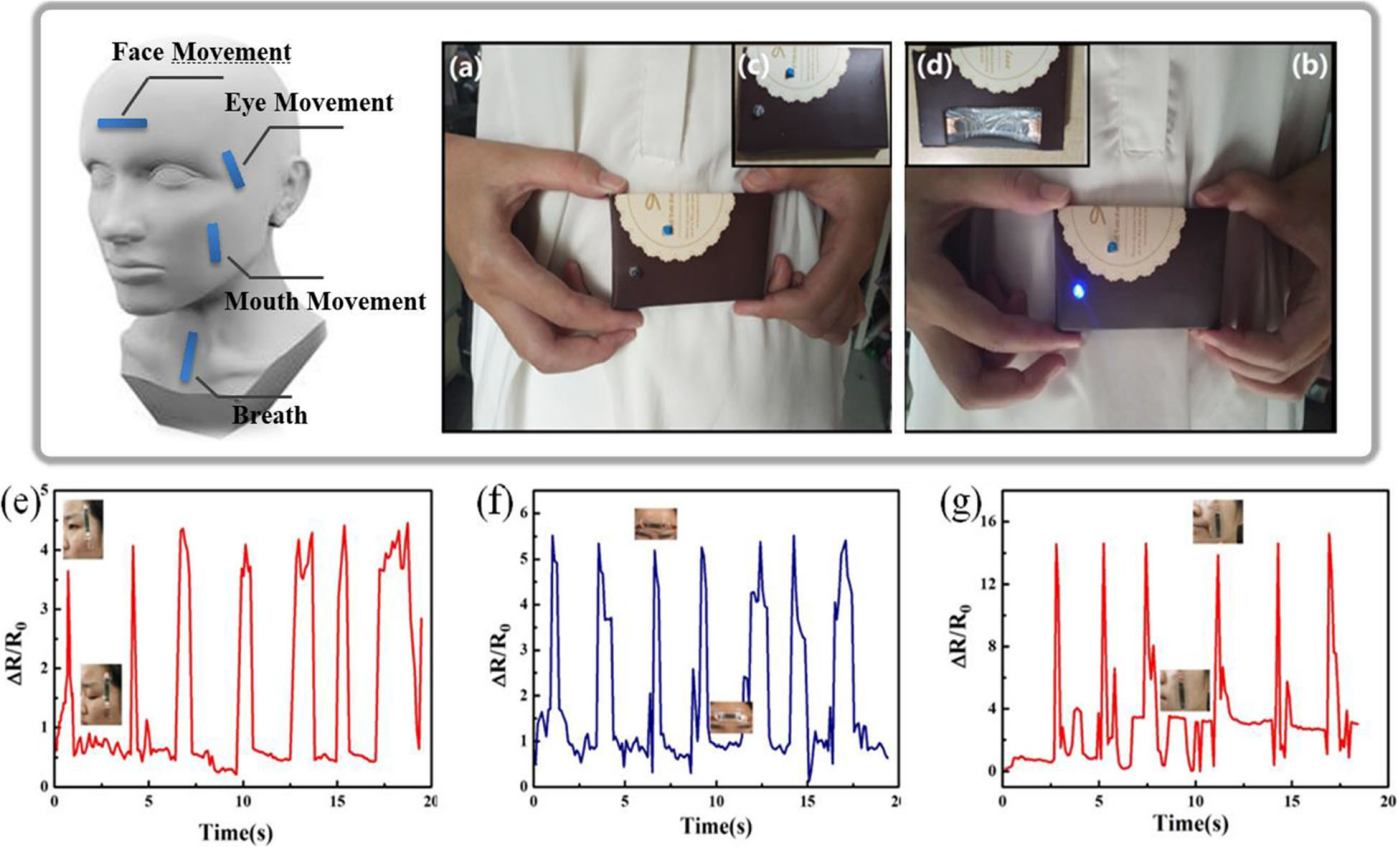
Portable breathing detector based on Ag@OH-f MWCNT soft strain sensor with wrinkled structure. **a** Breathing in and (**b**) breathing out. Pictures of the (**c**) top and (**d**) bottom sight from the portable breathing detector. **e** Facial, (**f**) frown, and (**g**) smile expression recognition of blink

In this study, we applied the sensing unit with high sensitivity and a relatively wide strain range that was developed in a portable breathing detector (Fig. 5). The working scene of the detector in monitoring the respiratory frequency is shown in Fig. 5a and b. The top and bottom views of the detector are shown in Fig. 5c and d, respectively. The sensor was stretched and the resistance increased when breathing in. As a result, the current was too low to light up the light-emitting diode (LED). In contrast, the LED was lit when air was breathed out. Furthermore, the relative resistance change was exploited in facial expression recognition, as shown in Fig. 5e to g. The relative resistance change of the sensor was 4 ± 0.2 when the volunteer blinked. When the same volunteer frowned, the relative resistance was identically changed to be 5.5 ± 0.1. Interestingly, the smiling action led to a relative resistance change as large as 15 ± 0.5. The results demonstrated that the Ag@OH-f MWCNT-based wrinkle structured sensor has great potential for a wide range of applications in healthcare sensing and human motion detecting.

Performances of different soft strain sensor materials are compared. As depicted in Table 1, compared with strain sensor based on other 1D nanomaterials, the wrinkle structured sensor based on OH-f MWCNTs exhibited good conductivity, improved stretchability, excellent gauge factor, and stability.

**Table 1:** Performance comparison of soft strain sensor materials

Materials	Stretchability	Linearity	Conductivity	Stability	Gauge factor
CNT	30–150%	Linear	Low	Good	1
AgNWs	70%	Linear up to 40%	High	Good	2–14
CuNWs	10%–250%	N/A	Low	Unstable	54.4
AuNWs	100%	Linear	Low	Good	20.4–61.4
ZnONws	2%	Linear	N/a	N/A	114
Platinum (Pt)	2%	Nonlinear	High	Unstable	2000
Hybride materials	140%–490%	N/A	High	N/A	24–95
This work	*42.2%*	Linear	High	Excellent	*412.32*

## Conclusion

In this paper, a high sensitivity and flexibility strain sensor consisting of PDMS and Ag@OH-f MWCNT was designed. Ag@OH-f MWCNT nanocomposites were prepared by reducing Ag ions along MWCNTs. The use of OH-f MWCNTs is essential for increasing the binding energy of Ag atoms and carbon nanomaterials to improve the stretchability of nanocomposites. At the same time, the oxygen plasma surface treatment of PDMS is important for manufacturing sensors with wrinkled structures to achieve a stable and unique sensing performance. The resistance and piezoresistive results show that the sensor has a GF of 412 and a strain range of 42.2%. The use of sensors in respiratory frequency testing and facial motion monitoring has demonstrated that well-designed sensors with new nanocomposites and wrinkled structures can be used in wearable devices for a multitude of purposes.

## Data Availability

The datasets supporting the conclusions of this article are included within the article (and its additional file(s)).

## Abbreviations

Ag@OH-f MWCNTs: Silver-coated hydroxyl-functionalized multiwall carbon nanotubes
AgNPs: Silver nanoparticles
CNTs: Carbon nanotubes
GF: Gauge factor
LED: Light-emitting diode
OH-f MWCNTs: Hydroxyl-functionalized multiwall carbon nanotubes
PDMS: Polydimethylsiloxane
SEM: Scanning electron microscope
TEM: Transmission electron microscopy
